# COVID-19: No Guaranteed Protection from Future Infection after the Initial Diagnosis

**DOI:** 10.1155/2021/6617719

**Published:** 2021-03-30

**Authors:** Christopher Chew, Supriya Mannepalli

**Affiliations:** Northeast Georgia Medical Center, 743 Spring Street, Suite 710, Gainesville, GA 30501, USA

## Abstract

The world of microbiology is vast in nature, and viruses continue to be a subset containing a lot of unknowns. Initial infection with certain viruses, such as varicella zoster virus and measles, allows for development of lifelong immunity; on the other hand, the influenza virus requires yearly vaccination, which may not provide adequate immunity. This can be attributed to antigenic shift and drift, rendering previously made antibodies ineffective against new strains of influenza. This article describes six cases of patients who presented with mild acute respiratory symptoms and tested positive for COVID-19 virus. After recovering from initial illness and being asymptomatic for several months, they developed recurrence of acute respiratory symptoms and, again, tested positive for COVID-19 virus, in more severe form than initial presentation. In the current state of the world, COVID-19 has created a lot of unknowns in the medical community, including patient presentation and treatment. COVID-19 research is evolving daily, but many questions remained unanswered. “Will a sufficient antibody response be created by the human body in those infected with COVID-19 and how long will that immunity last?” “Will antigenic drift occur quickly allowing the virus to evade previously made antibodies?” During initial surveillance of the COVID-19 virus, we were expecting development of an immune response comparable to SARS-CoV-1 and MERS-CoV, given the viral similarities. Unfortunately, based on our observations, this may not necessarily be true and will be further discussed in the presented article.

## 1. Background

The COVID-19 pandemic caught the medical community off guard with the amount of uncertainty regarding the virus. The treatment algorithms change frequently, and the basic understanding of how the virus acts is also evolving. Multiple theories have been put forward by the infectious disease community, with a frequent proposition stating infection with the COVID-19 virus results in adaptive immunity. Kirkcaldy et al. [1] presented the idea that antibodies are generated over subsequent weeks after initial infection, suggesting unlikely future reinfection with the same virus. With the ever-evolving information gathered about the COVID-19 virus, evidence implies adaptive immunity may not develop in all patients who have been infected by the virus, and antibodies may wean off in a short time period.

Not only are there a variety of theories behind the infection, but there have also been several tests developed over the past months for diagnosis of COVID-19. The first test employed by the emergency department (ED) at our facility was a send out nasal swab developed by CSI Laboratories. The test is a single-plex, real-time PCR, consisting of three processes in a single assay, including reverse transcription of target RNA to cDNA, PCR amplification of target and internal control, and simultaneous detection of PCR amplicons by fluorescent dye-labelled probes. This test is positive when the plasma contains at least 1000 copies of the virus. Cycle thresholds for a positive test are less than 40 N1, N2, and RNAseP. For a positive test, N1 and N2 must be positive, while RNAseP must be positive for a valid test. An inconclusive test is when either N1 or N2 is positive, while the other is negative.

Another test is a send out used by the local urgent care and the emergency department done by Diagnostic Solutions Laboratory (DSL). The laboratory used a PCR test with detection geared towards the genes N1 and N3, and spike gene. A positive test equates to all three genes being present in a cycle threshold less than 35. Additionally, RNAseP must be present for this test to be considered valid.

The COVID-19 test used primarily by our inpatient services at our facility is a nasal swab developed by ARUP Laboratories. This test done by PCR utilizes four different assays performed on 3 platforms including Thermo Fisher, Roche, and Hologic to detect the virus. The Thermo Fisher platform tests for Orf1ab/O-methyltransferase gene, N gene, and S gene with a cycle threshold of 37. The Roche platform tests for Orf1ab/O-methyltransferase and Env E-gene with a cycle threshold of 38. Hologic platform tests for Orf1ab/O-methyltransferase gene with a cycle threshold of 37.

The test designed by Quest Diagnostics, which was used for one of the patients in this article, is a PCR test designed to target the N1 and N3 proteins. The cycle threshold for this test is less than 40. The test requires both N1 and N3 proteins to be detected for a positive test, and it is inconclusive if only one protein is detected.

Two PCR tests within our hospital have been used; the first one is a PCR test developed by Cepheid. This test is a nasopharyngeal swab and tests for COVID-19, flu A, flu B, and RSV. Within the COVID-19 portion, the target is the N2 nucleocapsid gene and the envelope protein gene, E. The cycle threshold for this product is 45. Detection of the N2 and E genes equates to a positive test. The other test within our hospital is the multiplex PCR test developed by BioFire. For COVID detection, the test evaluates the spike protein, S, and the membrane protein, M. The test is positive if either or both proteins are detected, and its cycle threshold is 30.

## 2. Case Presentations

The first case is a 49-year-old male with no significant past medical history who initially had upper respiratory illness symptoms in February 2020 but was not diagnosed due to lack of testing ability at the time. In April, he presented to urgent care with nasal congestion and known exposure to individuals with COVID-19. At this time, his COVID-19 test was positive, but given nonspecific symptoms, he was not started on COVID-19-related treatments. The patient did not require oxygen supplementation, vital signs were stable, and hospitalization was not warranted. He was re-tested for COVID-19 five days later, both tests done by DSL, and tested positive again, while his only symptom of nasal congestion had already resolved. Serum IgG levels were negative six days after the initial positive test.

**Figure 1:**
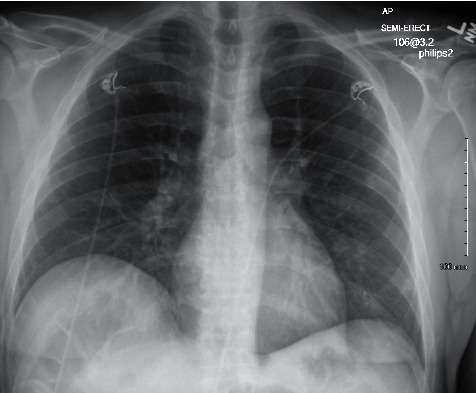
Case 1 CXR on 1^st^ admission.

**Figure 2:**
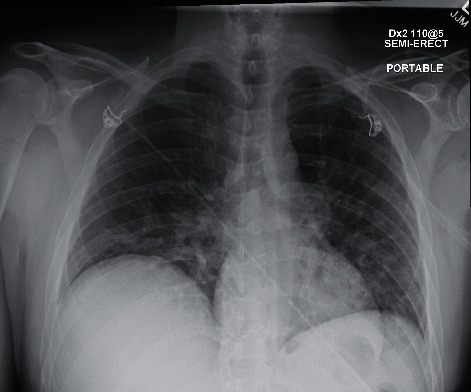
Case 1 CXR on 2^nd^ admission.

**Figure 3:**
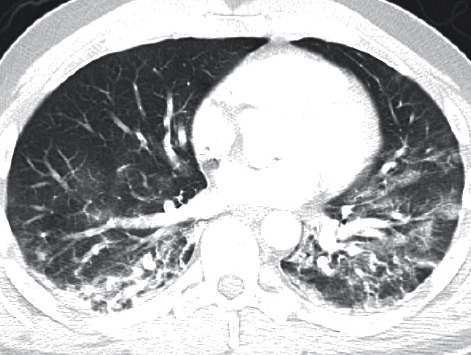
CT of the chest from 2^nd^ admission.

In July, he had a new exposure to a family member with a COVID-19 infection and presented to the emergency department with upper respiratory symptoms, including low-grade fever, shortness of breath, nausea, and diarrhea. He was tested for COVID-19 and was subsequently negative by the test done through CSI. ABG during this encounter resulted in a pH of 7.483, pCO_2_ 34.3, pO_2_ 69, and HCO_3_ 25.7. Imaging done at this time, as seen in Figure 1, did not show any significant airspace disease. Due to mild symptoms, he was discharged from the ED, as he did not require oxygen supplementation and treatment was deemed unnecessary. Four days later, he presented to the emergency department once again due to worsening shortness of breath and acute hypoxic respiratory failure. Imaging revealed worsening airspace disease on chest X-ray as seen in Figure 2 and CT imaging seen in Figure 3. ABG at this time resulted in a pH of 7.485, pCO_2_ 33.4, pO_2_ 66, and HCO_3_ 25.2. The patient required a maximum of 2 L of O_2_ supplementation via nasal cannula during this hospital stay. His COVID-19 test by CSI Laboratories was positive, the serum IgG was negative, and the stool PCR was positive. The patient was discharged once he was hemodynamically stable and no longer requiring oxygen. During this hospital admission, the patient received dexamethasone, tocilizumab, two units of convalescent plasma, and completed a five-day course of remdesivir. The SARS-CoV-2 antibody test done a couple of weeks after discharge resulted positive for serum IgG.

**Figure 4:**
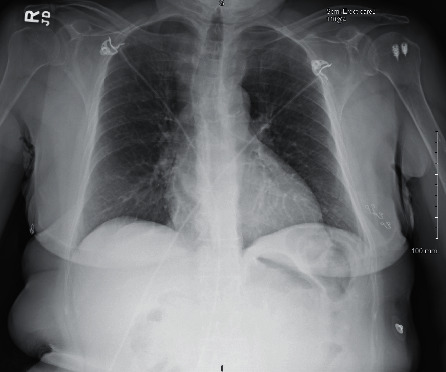
Case 2 CXR from 1^st^ ED visit.

The second case is a 73-year-old female with a past medical history significant for diabetes, hypertension, and GERD who initially presented to the ED with viral-like symptoms at the beginning of March 2020 but was not tested due to limitation of testing supplies at the time. Imaging conducted as seen in Figure 4 demonstrated no significant airway disease. Due to the mildness of her symptoms, including low-grade fever, saturation level greater than 95% on room air, and hemodynamic stability, she was discharged from the ED. The patient was instructed to follow up with her PCP outpatient, which she did at the end of April. During that visit, she was tested for COVID and, while asymptomatic, found to be positive on the DSL test. She subsequently tested negative two weeks later via DSL.

**Figure 5:**
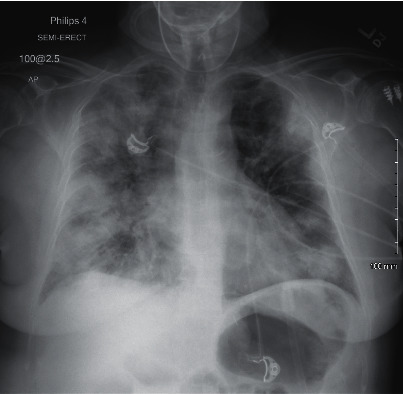
Case 2 CXR on admission from 2^nd^ infection.

**Figure 6:**
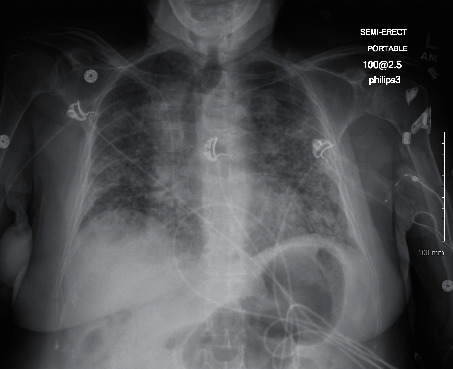
Case 2 CXR prior to electing for hospice care.

At the end of July, the patient was admitted to the hospital for increasing shortness of breath. Chest X-ray done on arrival, as seen in Figure 5, demonstrated significant airway disease concerning for ARDS. ABG on admission resulted with a pH of 7.362, pCO_2_ 74, HCO_3_ 18.4, and pO_2_ 74. She was started on dexamethasone and remdesivir and given one dose of tocilizumab for high suspicion for COVID-19. The COVID-19 PCR test subsequently returned positive by CSI. Her oxygen demand increased to BIPAP support as her hospital stay progressed. Throughout her stay, daily ABGs showed worsening hypoxia and pO_2_ decreased to 51, despite being on BiPAP support at 100% FiO_2_ and 40 L flow. At this time, the patient's family did not want intubation and elected for hospice care. Chest X-ray imaging done prior to the hospice transition is demonstrated in Figure 6 and showed worsening airspace disease. Unfortunately, the patient passed away three weeks after admission.

**Figure 7:**
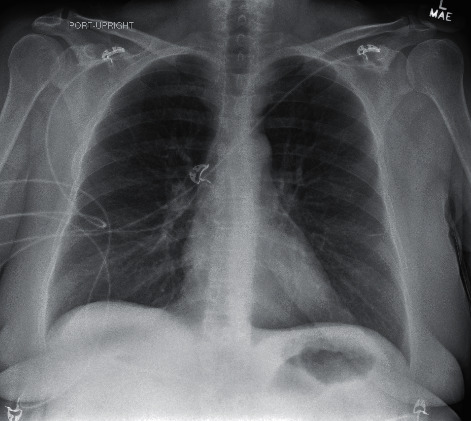
Case 3 CXR from first infection.

**Figure 8:**
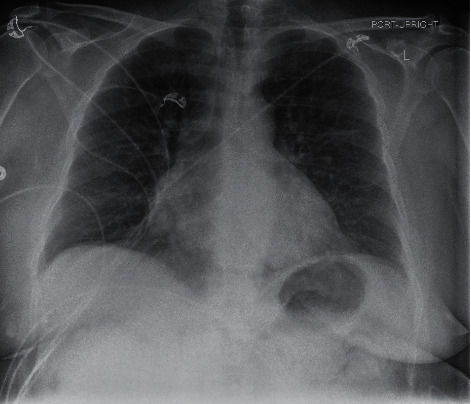
Case 3 CXR from July infection.

The third case is a 50-year-old female with a past medical history significant for COPD, CHF, and seizure disorder who was initially admitted at the end of March 2020 for a COPD exacerbation. Imaging done at this time, as seen in Figure 7, did not show any significant acute processes. At this time, her COVID-19 PCR test was negative by DSL. She presented to the emergency department in mid-April for a worsening cough and was tested once again for COVID-19, which was negative by DSL. A week later, she was admitted for worsening shortness of breath, wheezing, and worsening cough; now, she tested positive for COVID-19 via PCR by DSL and discharged two days later. During the short admission, the patient never required oxygen supplementation and was considered safe for discharge, due to mild symptoms and clinical diagnosis more indicative of acute COPD exacerbation, not requiring COVID-related treatments. One and a half weeks later, she presented to the emergency department again for worsening shortness of breath and discharged three days later; DSL COVID-19 test was negative at this time. She was admitted once again in June 2020 for three days of worsening shortness of breath secondary to a COPD exacerbation, and COVID-19 testing during this time was negative by DSL. At the beginning of July, she once again presented to the emergency department for worsening shortness of breath and was admitted for two days, testing positive for COVID-19 via PCR by CSI. Chest X-ray done during this admission was concerning for airspace disease, including pulmonary edema as seen in Figure 8. With concern for acute COPD and CHF exacerbations, the patient was started on pertinent treatments including diuretics, empiric clarithromycin, and IV steroids. The patient was discharged on a prednisone taper and clarithromycin. Due to the mildness of her respiratory symptoms, she was not started on COVID-related treatments. The patient once again never required oxygen supplementation during the short hospital stay.

**Figure 9:**
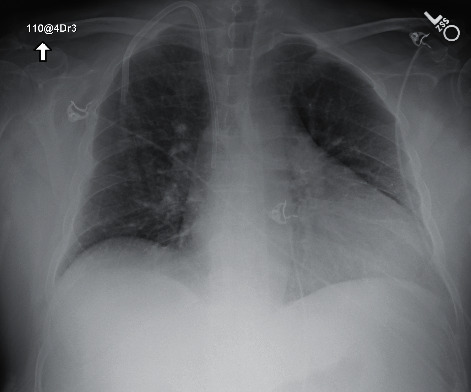
Case 4 CXR from 1^st^ infection on 1^st^ admission.

**Figure 10:**
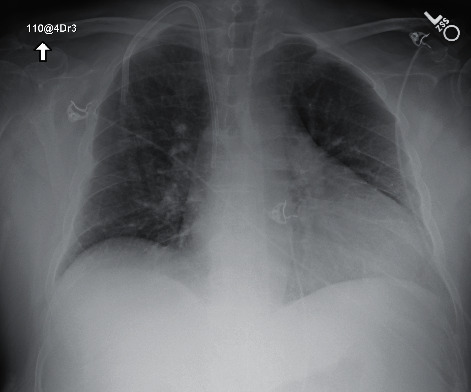
Case 4 CXR at the start of 2^nd^ admission for 2^nd^ infection.

The fourth case is an 80-year-old male with a past medical history significant for end-stage renal disease, hypertension, and diabetes who initially presented to the ED at the beginning of April 2020 with a chief complaint of general weakness. Chest X-ray done during this time, as seen in Figure 9, was concerning for airspace disease. ABG done on arrival demonstrated a pH of 7.466, pCO_2_ 40.4, pO_2_ 115, and HCO_3_ 28.7. He ended up testing positive for COVID-19 via PCR by DSL during this admission. He did not demonstrate any respiratory symptoms and did not require any oxygen supplementation during this admission. At the time, due to the limited understanding of the disease and treatment availability, the patient was initially treated with hydroxychloroquine, but the patient did not tolerate the medication due to QTc prolongation. He was discharged 4 days after admission, once deemed clinically stable and weaned off oxygen. The patient returned at the beginning of July for a malfunctioning vas-cath and tested negative for COVID-19 by DSL during this short stay. He returned to the hospital at the end of July for altered mental status and lethargy. Chest X-ray showed similar pattern as before when he was first diagnosed with COVID in April, as seen in Figure 10. During this hospital stay, he once again tested COVID-19 positive via CSI. The patient's ABGs are noted in Table 1. He required 2 L of supplemental oxygen four days into admission, but oxygen demand soon increased to 5 L via nasal cannula. Care was then escalated to BPAP, right before intubation. The ABG progression can be seen in Table 1.

**Figure 11:**
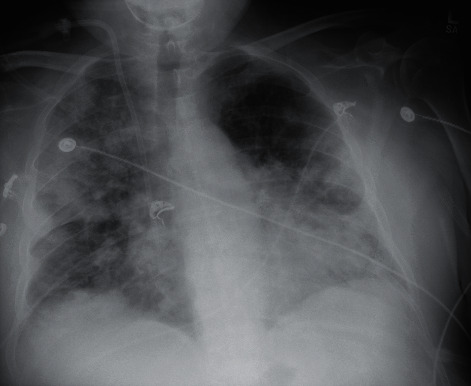
Case 4 CXR before intubation during 2^nd^ infection.

**Figure 12:**
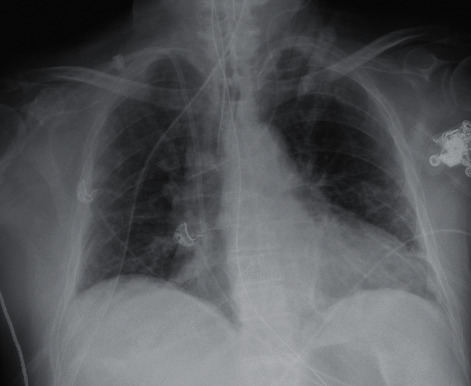
Case 4 CXR 4 days after intubation during 2^nd^ admission.

**Figure 13:**
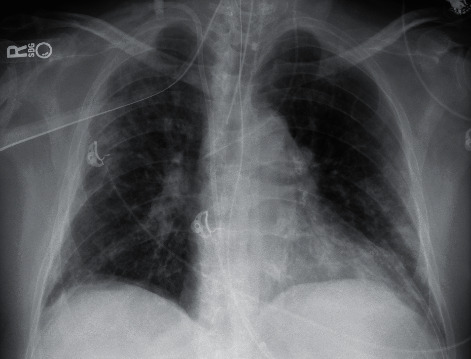
Case 4 CXR prior to death during 2^nd^ admission.

Chest X-rays seen in Figures 10–13 demonstrate the progression of the COVID-19 disease process during the patient's stay. During the length of the 16-day admission, the patient received empiric antibiotics, dexamethasone, vitamin C, and zinc.

**Figure 14:**
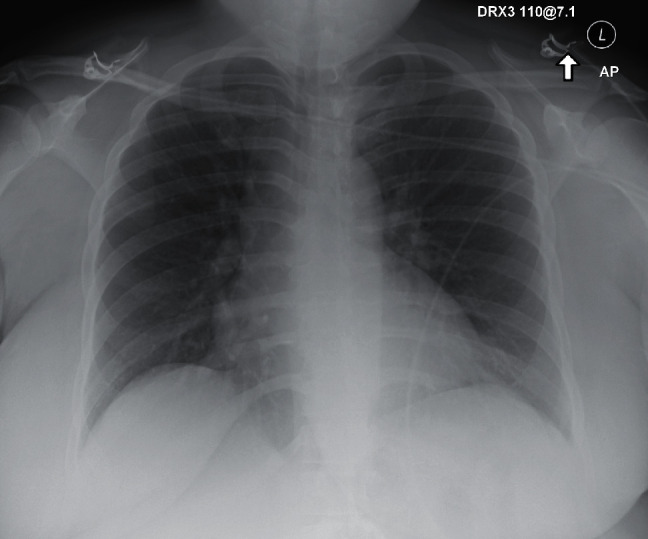
Case 5 CXR first ED visit in July.

**Figure 15:**
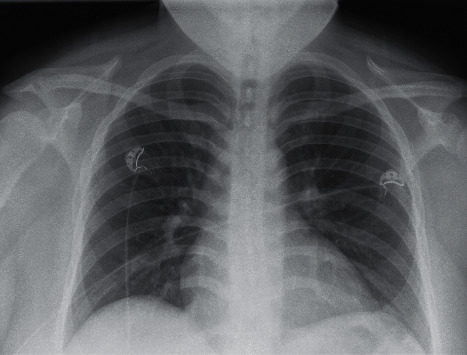
Case 5 CXR during 2^nd^ ED visit in July.

The fifth case is a 24-year-old female with a past medical history significant for asthma, morbid obesity, and PCOS who initially presented to urgent care at the end of April 2020 with complaints of fever, nausea, vomiting, and cough. She tested positive for COVID-19 via PCR by DSL, but had no signs of respiratory distress, and was medically stable for discharge home from the ED without need for hospitalization and COVID-19 treatment. At the beginning of July, she presented to the emergency department with upper respiratory symptoms and fever. She tested positive for COVID-19 via PCR by ARUP. Imaging done at this time, as shown in Figure 14, demonstrated no significant acute processes. Once again, she was medically stable with no signs of respiratory distress and was discharged home from the ED after 24 hours of observational monitoring, requiring no oxygen supplementation or COVID-19 treatments. The patient returned to the emergency department three weeks later at the end of July complaining of lower extremity weakness. She tested positive for COVID-19 via PCR by CSI. Chest X-ray done at this time demonstrated worsening infiltrates as seen in Figure 15. ABG done at the time of arrival demonstrated a pH of 7.401, pCO_2_ 38.6, pO_2_ 87, and HCO_3_ 23.5. The patient demonstrated no respiratory symptoms and did not require any oxygen supplementation at this time, despite being COVID-19 positive. CT imaging of the head and MRI of the spine were negative for acute findings. An LP was done, which was noncontributary. As the etiology of her lower extremity weakness was uncertain and the patient had a recent positive COVID-19 test, infectious disease was consulted and recommended treatment for COVID-19 with appropriate therapy, for a possible atypical presentation of COVID-19 virus. The patient was treated with a course of solumedrol and remdesivir. She was discharged 8 days after admission with a steroid taper. She later presented at a follow-up with neurology and was diagnosed with Guillain–Barré syndrome two weeks after being discharged from the hospital for COVID-19.

**Figure 16:**
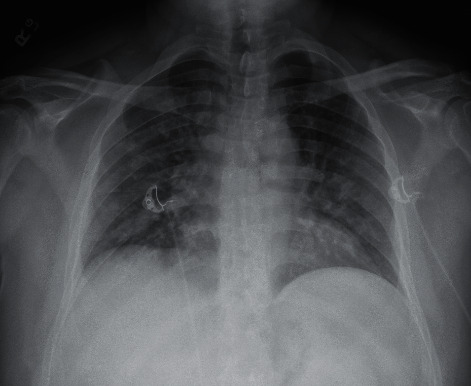
Case 6 CXR during November admission.

The final case is a 46-year-old male with a past medical history significant for hypertension and type 2 diabetes, who initially presented to an urgent care at the end of April for a cough, shortness of breath, and diarrhea over a two-week period. Given exposure history to COVID-19, he was tested for the virus and found to be positive via PCR by DSL. The patient was sent home from the urgent care facility, due to mildness of his respiratory symptoms, being hemodynamically stable, and not requiring pertinent COVID-19 treatments. He subsequently tested negative in May via PCR by DSL. The patient presented to the ED at the beginning of November due to a one-week history of persistent fever, myalgias, chest congestion, cough, and progressive shortness of breath. A day prior to going to the ED for worsening symptoms, the patient presented to an urgent care where COVID-19 testing was positive via PCR conducted by Quest Diagnostics. On arrival to the ED, the ABG performed demonstrated a pH of 7.404, pCO_2_ 33.5, pO_2_ 102, and HCO_3_ 33.5. Max O_2_ supplementation during this stay was 2 L of oxygen by nasal cannula. Chest X-ray on arrival, as seen in Figure 16, demonstrated severe bilateral airspace disease. During the admission stay of 3 days, the patient received remdesivir, dexamethasone, vit C, zinc, and empiric antibiotics and was subsequently discharged home.

## 3. Discussion

The purpose of this article is to demonstrate that the information regarding COVID-19 is ever-evolving. The initial belief of developing immunity from COVID-19 virus after infection is proving to be inaccurate. There is no genetic sequencing information to prove whether infections in these patients throughout their multiple illnesses were from the same or different strains; however, these clinical scenarios raise concern of new infection from COVID-19 after initial illness, and this cannot be ignored. Whilst it is hard to prove whether these cases represent a new infection versus reactivation, confirmed new exposure followed by development of symptoms in one of them is highly concerning for reinfection. Another concern for reinfection is the timing of subsequent negative tests, followed by a positive test after a significant amount of time.

As written by Kellam and Barclay [2], an antibody response is expected between 10 and 14 days after infection, but there is the possibility that, like other coronaviruses, the antibodies will decrease over time. There is also evidence through these cases that these individuals initially had mild courses of illness. It was their second infections that proved to be much worse and required hospitalization.

Ibarrondo et al. in their letter to the editor correspondence in the *New England Journal of Medicine* [3] state that while it is unknown how protective an adaptive immunity may be, the duration of the COVID-19 antibodies that exist may offer finite immunity. The antibodies generated start to trend down over one to three months after the initial diagnosis. This would allow a possible reinfection, due to a limited immune response from an adaptive immunity standpoint.

Long et al. [4] were able to detect the virus for up to four weeks after infection, showing the extended duration of the virus' presence. It was initially thought that the virus may behave similarly to the other coronavirus family viruses, such as SARS-CoV-1 and MERS-CoV, with IgG levels remaining for greater than two years after an infection, to provide immunity. Ultimately, it has been proven that the IgG levels in COVID-19-infected individuals decreased much faster than the other coronavirus family members, with a noticeable decrease within a few months after the initial diagnosis. Those with asymptomatic infection became seronegative earlier than those with symptomatic infection.

A recent study conducted by Tillet et al. [5] demonstrated genomic evidence of a 25-year-old man who was diagnosed with COVID-19 with a positive test in mid-April 2020, followed by two negative tests in May of 2020. A test done at the beginning of June 2020 upon presentation of viral symptoms was positive. Genetic sequencing done of the viral samples from the two different nasal swabs resulted in distinct genetic differences despite both being COVID-19. This report suggests that COVID-19 reinfection is very much possible.

While we hope that the development of herd immunity might be a strategy to prevent the spread of COVID-19, it can prove challenging if the immunity only lasts for two to three months after the initial infection. While we hope that COVID-19 infection will result in lifelong immunity, as seen with varicella zoster or measles, current evidence and our experience does not support this theory.

Prevention of influenza requires yearly vaccines, due to the minor genetic changes via antigenic drifts or occasional major changes due to antigenic shift the virus undergoes. The unknown of how COVID-19 behaves may prove difficult to generate vaccines to trigger an immune response. Vaccines will be challenging if there is in fact rapid antigenic drift occurring, which would explain why individuals can be reinfected in as little as two months after their initial infection. This supports the notion that reinfection with COVID-19 is possible and should be included in a differential in any patient that may be presenting with upper respiratory symptoms regardless of a prior diagnosed infection of COVID-19.

## Figures and Tables

**Table 1:** Significant ABGs during second hospital admission for Case 4.

Timing	pH	pO_2_	pCO_2_	HCO_3_	FIO_2_ (%)
On admission	7.386	77	40	23.5	21
Prior to intubation	7.377	59	22.9	13.1	40
After intubation	7.394	27.8	104	17	80
Two days after intubation	7.492	29.8	102	22.8	40
Four days after intubation	7.518	29.9	64	24.3	30
Morning prior to expiration	7.508	214.5	173	19.4	100
